# 

**Published:** 1871-09

**Authors:** 


### RUSH MEDICAL COLLEGE.

### FACULTY.

J. V. Z. BLANEY, A.M., M.D.; Prof. of Chemistry and Pharmacy.
JOS. W. FREER, M.D., Prof. of Physiology and Microscopic Anatomy.
J. ADAMS ALLEN, M.D., LL.D., Prof. of Principles and Practice of Medicine.
E. INGALS, M.D., Prof. of Materia Medica and Medical Jurisprudence.
DeLASKIE MILLER, M.D., Prof. of Obstetrics and Diseases of Women and Children.
R. L. REA, M.D., Prof. of Anatomy.
MOSES GUNN, A.M., M.D., Prof. of Principles and Practice of Surgery and Clinical Surgery.
EDWIN POWELL, M.D., Prof. of Military Surgery and Surgical Anatomy.
JOS. P. ROSS, M.D., Prof. of Clinical Medicine and Diseases of the Chest.
EDWARD L. HOLMES, M.D., Prof. of Diseases of the Eye and Ear.
CHARLES T. PARKES, M.D., Demonstrator.
F. L. WADSWORTH, M.D., Assistant to Prof. of Physiology.
E. FLETCHER INGALS, M.D., Assistant to Professor of Materia Medica.
C. T. FENN, M.D., Assistant to Professor of Obstetrics.
L. W. CASE, M.D., Assistant to Professor of Chemistry.

The Twenty-eighth Annual Course of Lectures will commence on Wednesday, Sept. 27th, 1871, and continue twenty weeks.

FEES.—Lectures, $55.00; Matriculation, $5.00; Dissection, $5.00; Hospital, $5.00; Graduation, $25.00.

Daily Clinics at the Dispensary, (except Sundays.) Surgical Clinics on Saturday afternoons, throughout the year, at which patients from the *country* and *city* are treated gratuitously.

Hospital Clinics are abundant and varied.

For Annual Announcement, or any information with reference to the College, address the Secretary.

**DR. DeLASKIE MILLER,**
518 Wabash Avenue, CHICAGO.

